# Anticataract Effect of the Aqueous Extract of the Flowers of *Aspilia africana* in Murine Model of Diabetic and Age-Related Cataracts

**DOI:** 10.1155/2023/7867497

**Published:** 2023-04-27

**Authors:** Adwoa Frema Amanfo, Samuel Kyei, Yaw Duah Boakye, Clement Osei Akoto, Justice Kwaku Addo, Kofi Oduro Yeboah, Newman Osafo

**Affiliations:** ^1^Department of Pharmacology, Faculty of Pharmacy and Pharmaceutical Sciences, College of Health Sciences, Kwame Nkrumah University of Science and Technology, KNUST, Kumasi, Ghana; ^2^Department of Optometry and Vision Science, University of Cape Coast, Cape Coast, Ghana; ^3^Department of Pharmaceutics, Faculty of Pharmacy and Pharmaceutical Sciences, College of Health Sciences, Kwame Nkrumah University of Science and Technology, KNUST, Kumasi, Ghana; ^4^Department of Chemistry, College of Science, Kwame Nkrumah University of Science and Technology, KNUST, Kumasi, Ghana; ^5^Department of Chemistry, University of Cape Coast, Cape Coast, Ghana

## Abstract

**Background:**

The use of *Aspilia africana* in traditional medicine for the management of ocular diseases has been reported in India and some indigenous communities of Africa. The aim of this study was to investigate the aqueous extract of the flowers of *A*. *africana* (AAE) as an anticataract remedy using murine models of diabetic and senile cataracts.

**Methods:**

Preliminary phytochemical screening of the extract, in vitro antioxidant assays, and in vitro aldose reductase inhibitory activity were performed. For anticataract investigations of the extracts, diabetic cataract was induced by galactose administration in 3-week-old Sprague Dawley rats. The evaluation of experimentally induced age-related cataract was performed by administering sodium selenite to 10-day-old rat pups.

**Results:**

The phytochemical analysis revealed the presence of alkaloids, tannins, flavonoids, glycosides, and saponins. In vitro aldose reductase inhibitory property of the extract on rat lenses revealed that the AAE inhibited the enzyme activity with IC_50_ of 12.12 *µ*g/ml. For the anticataract investigations, 30, 100, and 300 mg·kg^−1^AAE-treated rats recorded significantly low (*p* ≤ 0.0001) cataract scores compared to the negative control rats, indicating a delay in cataractogenesis from the second week of treatment in the galactose-induced cataractogenesis. Similarly, the treatment with AAE caused a significant reduction (*p* ≤ 0.0001) in cataract scores compared to the negative control rats in the selenite-induced cataractogenesis. Markers of lens transparency, such as aquaporin 0, alpha-A crystallin, and total lens proteins and lens glutathione levels, were significantly preserved (*p* ≤ 0.05–0.0001) in each cataract model after AAE treatment.

**Conclusion:**

The study established the anticataract potential of the aqueous extract of flowers of *A*. *africana* in murine models, hence giving scientific credence to its folkloric use in the management of cataract.

## 1. Introduction

Cataracts are considered a primary root cause of blindness worldwide and are responsible for almost fifty percent of the occurrence of blindness and visual impairment in Africa [1, 2]. In Ghana, cataract accounts for about 54.8% of blindness [2]. Together with other ocular disorders such as glaucoma, these disorders exert a significant disease burden globally, estimated at 61.4 million disability adjusted life years (DALY) representing 4.0% of total DALY [4, 5].

Galactose cataract model mimics cataract secondary to an underlying disease such as diabetes [6]. It has the merits of being stable and simple compared to the glucose cataract model, it is economical to meet all research needs, and it can rapidly mimic the pathological mechanism of diabetic cataract. Galactose-induced cataract develops rapidly, is reversible, and is commonly employed in investigating the mechanism of action of drugs in diabetic complications [7].

Selenite-induced cataract in suckling rats also mimics age-related cataract. Its merits include its ability to produce rapid and reproducible cataract in a short period [8]. It is a suitable model to study the basic mechanism of human cataract formation in that they are similar in their vesicular formation, increased calcium and insoluble protein levels, and decreased levels of glutathione and water-soluble proteins [9]. Signs of cataract induced by sodium selenite include the activation of m-calpain that causes elevated calcium levels in a cascade of biochemical processes. There are also increased insoluble protein and oxidative stress and decreased lens-soluble proteins such as *α* and *β*-*γ* crystallin [10, 11].

Although cataract-related blindness is reversible by surgical intervention, many, however, resort to using plant medicines due to fear of surgery or its high cost of access in Ghana [10, 13]. This has accounted for the low patronage of surgery with just about 523 persons out of 2000 opting for cataract surgery in Ghana according to Morny et al. [14]. Owing to this, many patients with cataract resort to alternate treatment options or no treatment at all. One such alternate source of treatment is the use of medicinal plants, some of which have been shown by several studies to possess anticataract activity [15].

Flowers of *Aspilia africana* (Persoon) C. D. Adams (local name “mfomfo” among the Ashantis in Ghana) are commonly used to manage cataract in Ghana and some West African countries [16–19]. However, the anticataract activity of this plant has not been verified in any scientific work. Hence, this study's aim was to investigate the anticataract activity of the aqueous extract of flowers of *A*. *africana* in murine models to ascertain the claim of its anticataract use in traditional medicine. There is also the potential for the identification of drug leads that could further be developed into cost-effective medicines for the treatment and prevention of cataract-associated blindness.

## 2. Materials and Methods

### 2.1. Plant Collection

The flowers of *A*. *africana* were collected from Asakraka-Kwahu in the Eastern Region of Ghana in November 2020 and dried at room temperature. The plant sample was validated by Dr. George Henry Sam in the Herbal Medicine Department, Kwame Nkrumah University of Science and Technology (KNUST). Specimen (KNUST/HMI/2022/F001) has been stored in the Department's Herbarium.

### 2.2. Plant Material Extraction

A quantity of 150 g of the dried flowers of *A*. *africana* was pulverized using a blender (37BL85 (240CB6), Waring Commercial, USA) into fine powder. The powdered *A*. *africana* flowers were then macerated with 3.5 L of distilled water for 72 hours. The extract was then filtered using a filter paper (Whatman No. 1). The filtrate was concentrated in a rotary evaporator (Rotavapor R-210, Buchi, Switzerland) at a temperature of 40°C after which the concentrate was evaporated to dryness in an oven at 50°C (Gallenkamp OMT Oven, Sanyo, Japan). The dried extract was collected and stored in an airtight container. The brownish solid mass collected (percentage yield 9.37%) was stored in the refrigerator at 4°C and reconstituted with distilled water when needed and was referred to as the *A*. *africana* extract (AAE).

### 2.3. Chemicals

Galactose was purchased from ACROS, New Jersey, USA; tropicamide ophthalmic solution (1%) was purchased from Alcon Laboratories South Africa (Pty) Ltd; rat enzyme-linked Immunosorbent Assay (ELISA) kits (Glutathione (GSH), Bicinchoninic acid (BCA), alpha-crystallin A chain (CRYAA) and aquaporin 0 (AQPO)) were purchased from Shanghai chemical limited, Shanghai, China; sodium selenite, ammoniacal alcohol, sulphuric acid, chloroform, dragendorff reagent, Mayer's reagent, sodium hydroxide, Fehling's solution A & B, lead acetate, ferric chloride, ammonia (liquid), hydrochloric acid, acetic anhydride, and 2,2-dipheny-1-picrylhydrazyl (DPPH) were purchased from Sigma Aldrich, Germany; quercetin was purchased from Double Wood LLC, USA; gallic acid was purchased from Reg-LABOGENS, India; and ascorbic acid was purchased from Brenntag, Ghana.

### 2.4. Animals

Three-week-old Sprague Dawley (SD) rats of both sexes were purchased from the Center for Scientific Research in Plant Medicine (CSRPM), Mampong-Akuapem, in the Eastern Region of Ghana. The rats were housed in polyacrylic coops with soft wood shavings as bedding at room temperature, in the Animal House School of Biological Sciences, University of Cape Coast (UCC). The rats were fed commercial pellet diet (Agricare Limited, Cape Coast, Ghana) and had access to water *ad libitum*. In addition, 10-day-old Sprague Dawley rat pups of either sex were obtained from the School of Biological Sciences Animal House, UCC, and housed together with their dams as described above.

### 2.5. Preliminary Phytochemical Screening

To detect the phytochemicals present in AAE, phytochemical screening was performed on the extracts as described by Harborne [20].

### 2.6. Aldose Reductase Inhibition Assay

#### 2.6.1. Isolation of Aldose Reductase

Unrefined aldose reductase (AR) enzyme isolation was done according to the procedure described by Lee [21]. In brief, fresh rat eyes were collected and quickly conveyed to the laboratory on ice at 0–4°C and stored in a −40°C freezer to keep all biological composition intact. Non-cataractous transparent lenses were isolated by extracapsular extraction, and a homogenate of the isolated lenses was made as 10% w/v in 0.1 M phosphate buffer (pH 7.0) was added and centrifuged at 5000 × *g* for 30 min at 4°C to obtain the supernatant. The supernatant derived after centrifugation was collected and kept as the enzyme suspension.

#### 2.6.2. Assessment of Aldose Reductase Activity

AR activity was assessed using the procedure described by Dongare et al. [22] with slight modifications. Aldose reductase breaks down surplus D-glucose into D-sorbitol with resultant change of reduced nicotinamide adenine dinucleotide phosphate (NADPH) into NADP+. The inception of NADP+ is documented at a wavelength of 340 nm for 3 minutes [23]. In this assay, the extract was disintegrated in phosphate-buffered saline (PBS). A test cuvette that contained freshly made lens homogenate (50 *µ*l), different concentrations of extracts or quercetin dissolved in PBS (10–1000 *μ*g/ml), 0.1 M phosphate buffer (50 *µ*l) (pH 7.0), and 0.03 mM NADPH (50 *µ*l) was read against a reference cuvette made up of all substances except for the substrate. The determination was made in triplicate.

The enzyme reaction began after 0.04 mM of D-xylose (50 *µ*l) (substrate) was added. The reduction in absorbance was measured at a wavelength of 340 nm for 3 minutes. The percentage inhibition of AR activity was calculated using the following relation:

(1) $$\begin{array}{c} \%\;inhibition\left[\frac{absorbance\;of\;control-absorbance\;of\;sample}{absorbance\;of\;control}\right]\times 100\text{.} \end{array}$$

### 2.7. Antioxidant Activity of AAE

#### 2.7.1. Total Flavonoid Content (TFC)

The aluminium chloride colorimetry assay was used, which determines the total quantities of flavonoids present in the crude aqueous extract of *A*. *africana* flowers, as reported by Chang et al. [24]. The reaction solution contained 0.5 ml of extract, 0.3 ml of 5% of NaNO_2,_ and 0.3 ml of 10% AlCl_3_. The reaction mixture was incubated at room temperature for 30 minutes. 2 ml of 1 mol/L NaOH was again added to the reaction mixture, and the absorbance was read at 415 nm. Quercetin was used as the positive control. A standard curve was obtained using quercetin. Total flavonoid capacity of the extract was deduced from the standard curve obtained. TFC was indicated as quercetin equivalent (QE) in mg/g of the extract.

#### 2.7.2. Total Phenolic Content (TPC)

To find the gross amount of phenolic substances in the crude aqueous extract of *A*. *africana* flowers, the Folin–Ciocalteu method was used [25]. A mixture made of 0.1 ml Folin–Ciocalteu (0.5 N) and 0.5 ml of extract was incubated for a quarter of an hour at room temperature. 2.5 ml of NaHCO_3_ 2% (w/v) was added to the reaction mixture and again incubated at ambient temperature for an hour and a half minute. The absorbance was read at 760 nm. Gallic acid was utilized as the positive control. A standard curve was obtained using gallic acid. Total phenolic capacity of the extract was deduced from the standard curve obtained. TPC was indicated as gallic acid equivalent (GAE) in mg/g of the extract.

#### 2.7.3. Total Antioxidant Capacity (TAC)

To estimate the antioxidant capacity of the extract, a solution that contained 1 ml of extract and 3 ml of test reagent (0.6 M sulphuric acid, 28 mM disodium phosphate, and 4 mM ammonium molybdate) was incubated at 95°C for an hour and 30 minutes. The absorbance of this reaction mixture was read at 695 nm after it cooled down. A standard curve was obtained using gallic acid. The total antioxidant capacity of the extract was deduced from the standard curve obtained. TAC was determined as ascorbic acid equivalent (AAE) in mg/ml of the extract [26].

#### 2.7.4. Free Radical Scavenging Property

The scavenging power of DPPH by the extract was determined using the method described by Govindarajan et al. [27] and Sharma and Bhat [28]. To 3 ml of methanol, DPPH was added to produce a solution of 20 mg/l and 1 ml of the extract (2000–62.5 *µ*g/ml in methanol) was also added. The absorbance of the mixture was measured at 517 nm after incubation in the dark at 25°C for a 30-minute period. A milliliter of methanol (absolute) was added to 3 ml DPPH solution, incubated at room temperature for half an hour, and used as the blank. Vitamin C (100–0.78 *µ*g/ml) was utilized as the standard drug. It was observed that the absorbance reduced when free radical scavenging ability was increasing. Then, % inhibition was plotted against concentration, and the IC50 was estimated. The determinations were made in triplicate. The percentage (%) DPPH scavenging effect (% of the control) of the antioxidant was carried out as follows:

(2) $$\begin{array}{c} \%DPPH\;scavenging\;effect=\left[\frac{\left(AC-AE\right)}{AC}\right]\times 100\%\text{,} \end{array}$$

where AC = absorbance of the control and AE = absorbance of the extract.

### 2.8. Galactose-Induced Cataract Assay

The study used the method as described by Kyei et al. [29] with some modifications. 3-week-old Sprague Dawley rats were placed in five groups (*n* = 5). Four out of the five groups in each investigation received 3000 mg·kg^−1^ galactose *per os, b.d*. accompanied by treatment with 30, 100, or 300 mg·kg^−1^ of AAE, or 10 ml·kg^−1^ distilled water orally every day for 6 weeks. The fifth group of rats served as the normal control (NC) group that received neither galactose nor extract but was administered 10 ml·kg^−1^ of distilled water over the same experimental period.

Before the start of the study, rats in all groups had their crystalline lenses assessed for cataractogenesis, that is, the presence of vacuoles, and partial or total opacification of the lens, with the aid of a Magnon Slit Lamp (Model SL-250, Serial 12446, BOC Instruments, Japan). Subsequently, the lenses were examined weekly and any occurrence of cataract was graded and scored on a scale of 0–5, as a result of the extent of the opaqueness, as detailed by Sippel [30] (Table 1).

#### 2.8.1. Blood Sugar Determination

After an overnight fast, the initial blood sugar levels of every rat in all study groups were determined by expression of blood from the rats' vein in the tail making use of sugar measuring test strips and a glucometer (Accu Chek Performa, Roche Diagnostics, USA). Subsequently, the blood sugar level was determined weekly for six weeks before each slit-lamp assessment for cataractogenesis.

#### 2.8.2. Rat Body and Lens Weight Assessment

Rats induced with galactose cataract weights were checked before the study started and every week for 6 weeks. At the end of the study, the animals were weighed for their final body weights. The animals were euthanised, and their lenses were extracted extracapsularly and weighed. The extracted lens weight to the whole-body weight ratio was calculated using the following formula:

(3) $$\begin{array}{c} lens\;weight\;to-body\;weight\;ratio=\left[\frac{lens\;weight}{body\;weight}\right]\times 100\%\text{.} \end{array}$$

#### 2.8.3. Total Lens Protein Determination

A bicinchoninic acid protein ELISA test kit (Shanghai Chemical Ltd, Shanghai, China) was used to determine protein levels in the lens according to the manufacturer's protocol.

#### 2.8.4. Lens GSH Determination

The total lens glutathione concentration was determined using a glutathione ELISA kit (Shanghai Chemical Ltd, Shanghai, China). Lenses that were extracted were weighed and homogenised with PBS (pH 7.0). Then, they were centrifuged at 5000 × *g* for 30 minutes. The supernatant was transferred into Eppendorf tubes and used for this assay according to the manufacturer's protocol. The optical densities (ODs) of all the wells were read with a microtiter plate reader (URIT Medical Electronic Co., Ltd., Guangxi, China) at 450 nm. All determinations were done in duplicate.

### 2.9. Selenite-Induced Cataract Assay

The study used the method described by Kyei et al. [31]. In brief, 10-day-old rat pups were injected with sodium selenite (Na_2_SeO_3_) (15 *µ*mol/kg) dissolved in distilled water every day for 2 days subcutaneously. After the shot, the animals were grouped into five (*n* = 4, but 8 eyes each). Groups I–IV in each investigation were given 30, 100, and 300 mg·kg^−1^ of AAE or 10 ml·kg^−1^ distilled water orally, 30 minutes following the first sodium selenite shot. After this, treatment was carried out in a 12-hour interval for 21 days. Group V, which was the normal control, took neither selenite shots nor any treatment and was kept under similar housing conditions as the other pup groups. Following the 21^st^ day after the selenite shot, the pups' pupils were dilated using a tropicamide ophthalmic solution (1%). The evaluation of the lens for cataractogenesis was done using a Magnon Slit Lamp (Model SL-250, Serial 12446, BOC Instruments, Japan). Emerging cataracts were scored and graded.

#### 2.9.1. Grading and Scoring of Cataract

Cataract grading was done as by two independent assessors as represented in Table 2. The grades were scored, and percentage cataract score was determined using the following relation:

(4) $$\begin{array}{c} \%\;cataract=\frac{number\;of\;cataractous\;eyes\;per\;group}{total\;number\;of\;eyes\;per\;group}\times 100\%\text{.} \end{array}$$

#### 2.9.2. Determination of Lens Aquaporin 0 Levels

The manufacturer described the protocol for this assay. The concentration of aquaporin 0 in the lens was estimated using an AQP0 ELISA test kit (Shanghai Chemical Ltd, Shanghai, China). Lenses that were enucleated were weighed and homogenised with PBS (pH 7.0). Then, they were centrifuged at 5000 × *g* for half an hour. The supernatant was collected in Eppendorf tubes and used for this assay. The standards were constituted according to the manufacturer's directives. All other protocols were followed as stated by the manufacturer. ODs of each well were determined with a microtiter plate reader (URIT Medical Electronic Co., Ltd., Guangxi, China) at 450 nm. Each determination was made in duplicate.

#### 2.9.3. Determination of Soluble Protein Levels (CRYAA)

Total CRYAA concentration of the lens was estimated using a CRYAA ELISA test kit (Shanghai Chemical Ltd, Shanghai, China). The supernatant of the homogenised lens was subjected to a similar treatment as described previously.

#### 2.9.4. Histopathological Evaluation

The harvested lenses from the pups' eyes were fixed in 10% phosphate-buffered paraformaldehyde and wax-infiltrated with paraffin. Embedded tissues were thin-sectioned using microtome cutting machine (LAB Comercial, Barcelona, Spain), stained using haematoxylin and eosin dye [32], and fixed on glass slides for histopathological assessment.

### 2.10. Statistical Analysis

Statistical data analysis was performed using GraphPad Prism Version 8.0.1 (GraphPad Software, Inc., USA). The difference in treatment groups and controls was determined using one-way or two-way analysis of variance (ANOVA) and then Dunnett's multiple comparisons test (*post hoc* test). Values were considered statistically significant at *p* ≤ 0.05.

## 3. Results

### 3.1. Phytochemical Screening

As shown in Table 3, tannins, alkaloids, triterpenoids, flavonoids, glycosides, and saponins were present in AAE.

### 3.2. Aldose Reductase Inhibition Assay

AAE was tested for its effect on AR using an *in vitro* assay. The IC_50_ value for AAE in this study was compared to a standard AR inhibitor, quercetin, at concentrations of 50, 100, 200, 400, and 800 *µ*g/ml (Table 4). AAE showed inhibitory activity on aldose reductase (IC_50_ value of 12.12 *µ*g/ml).

### 3.3. Antioxidant Assay

**Figure 1:**
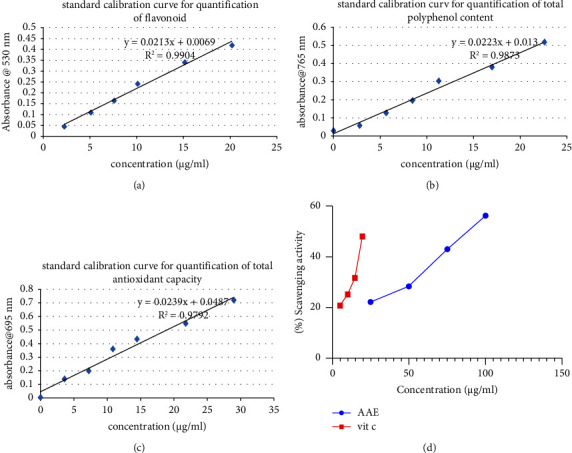
Standard calibration curve for flavonoid (a), total phenol (b), and antioxidant (c) quantification and free radical scavenging activity (d) of AAE.

In the antioxidant capacity assay of the extract, the total antioxidant capacity of quercetin, gallic acid, and ascorbic acid was found to increase with increasing concentration of the extract. The extract was established to have significant scavenging activity in a dose-dependent manner (Figure 1) with standard ascorbic acid, gallic acid, and quercetin equivalent determined to be 843.30, 764.73, and 408.90 *µ*g/g, respectively (Table 5).

### 3.4. Galactose-Induced Cataractogenesis

#### 3.4.1. Effect of AAE on Blood Sugar Level

**Figure 2:**
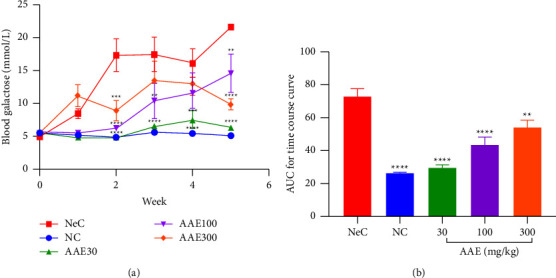
Effect of A. africana aqueous extract on (a) the time course curve and (b) the area under the time-dependent curve (AUC) of blood galactose levels in Sprague Dawley rats in galactose-induced cataract. Values are means ± SEM (n = 5). ^*∗∗*^*p* ≤ 0.01, ^*∗∗∗*^*p* ≤ 0.001, ^*∗∗∗∗*^*p* ≤ 0.0001; significance between the negative control group (NeC) and the other treatment groups.

This *in vivo* study evaluated the ability of AAE to lower blood sugar levels.  Figure 2(a) shows the times course curve, and the normal control (NC) rats showed a steady blood sugar (galactose) concentration of about 5 mmol·l^−1^ over the 5-week period. However, negative control rats (NeC) that received galactose only had a persistent increase in blood sugar (galactose) concentrations that reached about 22 mmol·l^−1^ during the fifth week after galactose ingestion.

Cumulatively, galactose administration in the negative control significantly (*p* < 0.0001) increased the plasma galactose concentration with a mean blood galactose concentration of 72.59 ± 4.996 compared to 26.32 ± 0.5524 of the normal control (Figure 2(b)). Giving rats AAE 30, 100, and 300 mg·kg^−1^ significantly (*p* < 0.0001) reduced the blood galactose concentration to 29.67 ± 1.712, 43.51 ± 4.652, and 54.27 ± 4.306, respectively, compared to the negative control.

#### 3.4.2. Effect of AAE on Galactose-Induced Cataractogenesis

**Figure 3:**
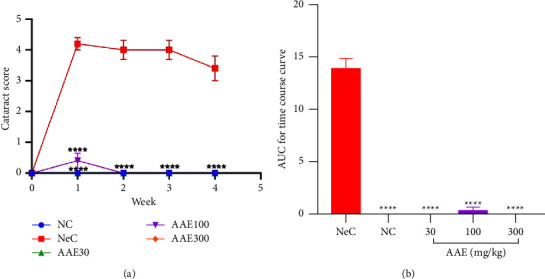
Effect of A. africana aqueous extract on (a) the time course curve and (b) the area under the time-dependent curves (AUC) of cataractogenesis in Sprague Dawley rats with galactose-induced cataract. Values are means ± SEM (n = 5). ^*∗∗∗∗*^*p* ≤ 0.0001; significance between the negative control (NeC) and the other treatment groups.

In the time course curve in Figure 3(a), it was observed that the normal control rats did not show any signs of cataract development over the 5-week period. However, the negative control rats that were given galactose only developed cataract that peaked in the 1^st^ week of galactose ingestion, with a slight drop but significantly (*p* < 0.05) high cataract score from the 2^nd^ week to the 4^th^ week. The total cataract score measured as AUC showed that the normal control (NC) had a mean cataract score of 0.000 ± 0.000; upon galactose administration to rats in the negative control (NeC), the mean cataract score increased to 13.90 ± 0.9798. Treatment of rats with AAE (30, 100, and 300 mg·kg^−1^) yielded a mean score of 0.000 ± 0.000, 2.200 ± 0.6633, and 0.000 ± 0.000, respectively (Figure 3(b)). Hence, the delay in cataractogenesis as compared to the NeC was significant (^*∗∗∗∗*^*p* ≤ 0.0001).

#### 3.4.3. Lens to Body Weight Ratio

**Figure 4:**
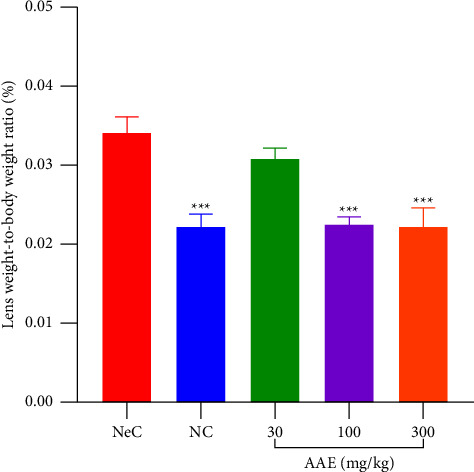
Effect of *A. africana* on lens to body weight ratio of Sprague Dawley rats with galactose-induced cataract. NeC = negative control. Each treatment was performed simultaneously with galactose (3000 mg·kg^−1^) b.d except for the normal control (NC). Values are expressed as mean ± SEM (*n* = 5). Comparison between the NeC and treatment groups: ^*∗∗∗*^*p* ≤ 0.001.

A physical representation of cataract is the presence of body weight loss and increase in lens weight or size after it has been enucleated [29]. When lenses were enucleated at the end of the study, it was observed that normal control rats had a normal lens weight, making their cumulative ratio less and significant (*p* ≤ 0.001) as compared to NeC (Figure 4). Negative control rats showed a notable increase in lens weight and a notable loss of body weight, causing the cumulative ratio to increase as well. In galactose challenged rats that were given AAE at 30 mg·kg^−1^, there was no significant change (*p* ≤ 0.05) in lens to body ratio. However, rats given 100 and 300 mg·kg^−1^ of AAE exhibited a significant reduction (*p* ≤ 0.001) in the lens to body weight ratio (Figure 4).

#### 3.4.4. Effect of AAE on Lens Glutathione (GSH) Levels

**Figure 5:**
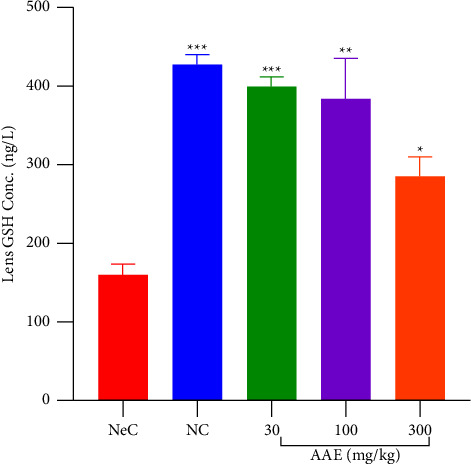
Effect of *A*. *africana* on lens GSH levels of Sprague Dawley rats with galactose-induced cataract. NeC = negative control. Each treatment was performed side by side with galactose (3000 mg·kg^−1^) b.d except for normal control (NC). Values are expressed as mean ± SEM (*n* = 5). Comparison between NeC and treatment groups: ^*∗*^*p* ≤ 0.05; ^*∗∗*^*p* ≤ 0.01; ^*∗∗∗*^*p* ≤ 0.001.

Normal control rats expressed high levels of GSH (427.8 ± 12.49 ng·l^−1^) which was significant (*p* ≤ 0.001) as against levels (160.4 ± 13.71 ng·l^−1^) achieved with rats in negative control. There was a significant (*p* ≤ 0.05–0.001) increase in the levels of glutathione in rats treated with AAE (30-300 mg·kg^−1^) when compared with negative control rats. There were 400.5 ± 12.33 ng·l^−1^, 384.7 ± 51.01 ng·l^−1^, and 286.9 ± 23.50 ng·l^−1^ of GSH recorded for rats treated with 30, 100, and 300 mg·kg^−1^ of AAE, respectively, (Figure 5).

#### 3.4.5. Effect of AAE on Lens Total Protein Levels

**Figure 6:**
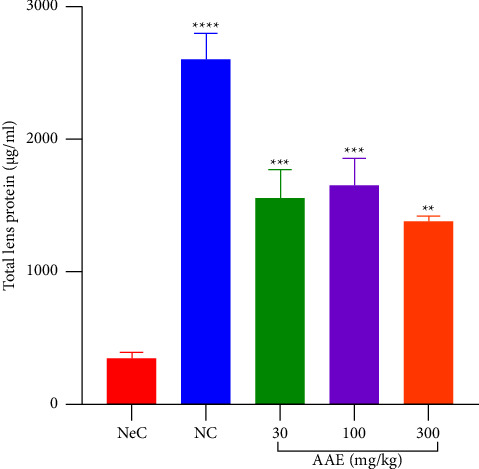
Effect of *A*. *africana* on the total protein levels of lens of Sprague Dawley rats in galactose-induced cataract. NeC = negative control. Each treatment was performed simultaneously with galactose (3000 mg·kg^−1^) b.d, except for normal control (NC). Values are expressed as mean ± SEM (*n* = 5). Comparison between NeC and treated groups: ^*∗∗*^*p* ≤ 0.01; ^*∗∗∗*^*p* ≤ 0.001; ^*∗∗∗∗*^*p* ≤ 0.0001.

Total protein levels in the lens of rats were estimated with a bicinchoninic test kit. In this study, total protein levels were determined in the presence or absence of AAE. There was a significant increase (*p* ≤ 0.0001) in total lens protein from normal control rats (NC) (i.e., 2609 ± 186.1 *µ*g/ml) as against the negative control group that had very low BCA levels (347.2 ± 44.10 *µ*g·ml^−1^). There was a significant (*p* ≤ 0.01) increase in the levels of total proteins in rats at all doses of AAE studied when compared with the negative control. 1561 ± 210.3 *µ*g·ml^−1^, 1656 ± 196.5 *µ*g·ml^−1^, and 1374 ± 43.96 *µ*g·ml^−1^ of total protein was obtained for rats treated with 30, 100, and 300 mg·kg^−1^ of AAE, respectively, (Figure 6).

### 3.5. Selenite-Induced Cataractogenesis

#### 3.5.1. Effect of AAE on Selenite-Induced Cataractogenesis

The eyes of these animals (pups) were observed for cataractogenesis by dilating them with 1% tropicamide solution before examination with a slit lamp to grade the cataract. In the slit lamp assessment, the normal control rats expectedly showed no cataract development. However, the negative control rats exhibited grade IV cataract development in 6 out of 8 eyes assessed (75%) with the remaining 2 eyes exhibiting grade III cataract development.

**Figure 7:**
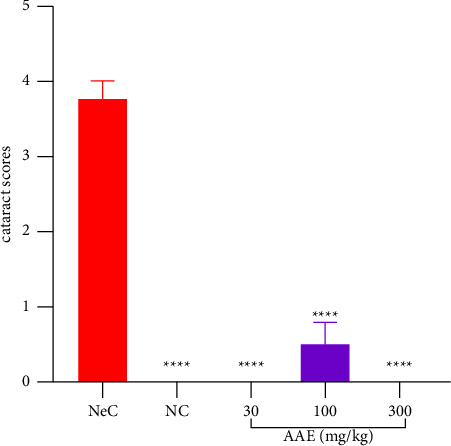
Effect of *A. africana* on the cataract score in Sprague Dawley rat pups with selenite-induced cataract. Values are expressed as mean ± SEM (*n* = 4). Comparison between the NeC and treated groups: ^*∗∗∗∗*^*p* ≤ 0.0001.

For AAE treated animals, AAE 30 mg·kg^−1^ treated rats had none of the 8 eyes developing cataract. Pups treated with 100 mg·kg^−1^ AAE had 3 (37.5%) out of the 8 lenses developing grade I cataract and the remaining 5 (67.5%) had no cataract. However, none (0%) of the lenses of the pups treated with 300 mg·kg^−1^ of AAE developed cataract. The cataract scores depicted that all AAE doses used alleviated cataract induced by Na_2_SeO_3_ significantly (*p* ≤ 0.0001) (Figure 7).

#### 3.5.2. Effect of AAE on Lens Soluble Protein Concentration (*α* Crystallin) (CRYAA)

**Figure 8:**
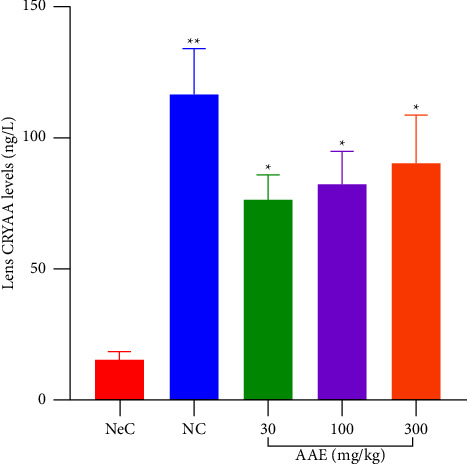
Effect of *A. africana* on lens soluble protein concentration of Sprague Dawley rat pups with selenite-induced cataract. Values are expressed as mean ± SEM (*n* = 4). Comparison between NeC and the treated groups: ^*∗*^*p* ≤ 0.05; ^*∗∗*^*p* ≤ 0.01.

The normal control pups had increasing levels of CRYAA (116.4 ± 17.69 ng·l^−1^) which were significantly high (*p* ≤ 0.01) as compared to CRYAA levels in the NeC (15.43 ± 3.005 ng·l^−1^). When animals were treated with AAE, it was observed that treated rats expressed higher levels of CRYAA which were significant (*p* ≤ 0.05) when compared to negative control (NeC) pups. In the extract treated rats, 76.28 ± 9.499 ng·l^−1^, 82.56 ± 12.26 ng·l^−1^, and 90.47 ± 18.10 ng·l^−1^ of CRYAA were recorded at 30, 100, and 300 mg·kg^−1^ dose levels, respectively. All extract-treated groups expressed significantly higher levels of CRYAA when compared to the NeC (Figure 8).

#### 3.5.3. Effect of AAE on Lens Aquaporin 0 (AQP0)

**Figure 9:**
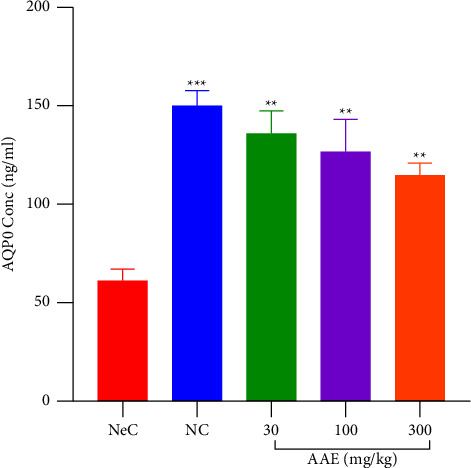
Effect of *A*. *africana* on the levels of aquaporin 0 in the lens of Sprague Dawley rat pups with selenite-induced cataract. Values are expressed as mean ± SEM (*n* = 4). Comparison between NeC and treatment groups: ^*∗∗*^*p* ≤ 0.01; ^*∗∗∗*^*p* ≤ 0.001. NeC, negative control.

In this study, the level of AQP0, a measure of the integrity of the lens water channel, and the ability to bind cells in the lens together were determined in the presence of AAE. It was observed that the normal control rats had increasing levels of AQP0 (149.8 ± 7.860 ng/ml) when compared to the negative control cataractous rats which caused a significant decrease to 61.34 ± 5.559 ng/ml in AQP0 recorded. Treatment with AAE (30–300 mg·kg^−1^) significantly (*p* ≤ 0.01) increased AQP0 levels compared to the vehicle treated group. The levels of AQP0 were determined to be 135.7.0 ± 11.91 ng/ml, 126.9 ± 16.31 ng/ml, and 115.2 ± 5.684 ng/ml in rats at doses of 30, 100, and 300 mgkg-1 of AAE, respectively. AQP0 levels in all treated groups were markedly high (*p* ≤ 0.01) when compared to the NeC (Figure 9). This implies that water channels in the lens are intact as compared to the NeC control pups.

#### 3.5.4. Histopathological Assessment and Analysis

**Figure 10:**
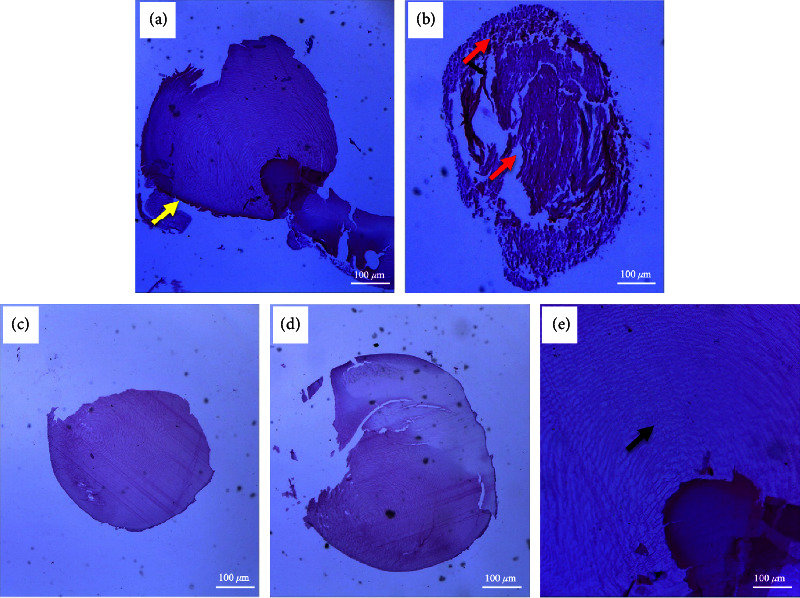
Photomicrographs of lenses: (a) normal lenses (normal control) showing a regular arrangement of lens fibres in the lens cortex; (b) negative control lenses showing an eroded epithelial margin and distorted lens fibre morphology within the cortex with fragmentation and gaps within the lens fibres; (c) selenite-induced lens cataract treated with 30 mg/kg AAE showing a fine arrangement of lens fibres interspersed with nuclei and also showing remnants of the epithelial layer; (d) selenite-induced lens cataract treated with 100 mg/kg AAE showing normal arrangement of transparent lens fibres; (e) selenite-induced lens cataract treated with 300 mg/kg AAE showing a normal arrangement of transparent lens fibres. The micron bar represents 100 *μ*m.

Histopathological images in all extract-treated rats showed that the integrity of the lens, as well as the normal architecture of the lens fibres, was preserved, as well as in normal control rats (Figures 10(b), 10(c), and 10(e)). Nonetheless, lens epithelial erosion and abnormal morphology of lens fibres were observable in the negative control (Figure 10(b)).

## 4. Discussion

The current study sought to investigate the anticataract property of *A*. *africana* flowers in galactose- and selenite-induced cataracts in three-week-old and ten-day-old Sprague Dawley rats, respectively. In the galactose model, a high consumption of galactose leads to traces of sugar in peripheral blood that contributes to the development of cataracts. In selenite-induced cataract, excess shots of the selenite salt cause cataractogenesis by insolubilization of soluble proteins (*α* and *β*-*γ* crystallin) [33] and distortion of calcium homeostasis [34] which will increase the levels of calcium and also increase oxidative stress [33].

Hyperglycemia associated with high consumption of galactose leads to the upregulation of aldose reductase [34], the enzyme implicated in the polyol pathway causing galactose to be metabolize into galactitol [37]. Galactitol builds up in the lens because it cannot cross the lens membranes by diffusion, leading to increased osmotic pressure of the lens and swelling of the lens and an increase in weight [38]. Additionally, there is an increase in the expression of reactive oxygen species that can also destroy the lens through oxidative stress [39].

Unlike glucose, galactose has a higher affinity with aldose reductase; besides, galactitol (alcohol metabolite of glucose) is more difficult to metabolize by sorbitol dehydrogenase than sorbitol (alcohol metabolite of glucose), and hence galactosemia is likely to produce more severe cataract in shorter periods [40–43]. Despite all these, the extract was able to reduce sugar levels, thereby averting hyperglycemia to prevent cataract development.

Extract treatment resulted in zero or low cataract scores recorded in cataract rats. This could be attributable to the aldose reductase inhibitory effect of the extract and the extract preventing possible oxidative stress triggered by ROS accumulation when galactitol builds up in the lens. This is further supported by the absence of the physical sign of cataract such as the loss of body weight and increase in lens weight [44].

There are several antioxidants in the lens, glutathione being the most abundant and vital for maintaining lens transparency [45, 46]. Additionally, a sign of precataractous change is the reduction of total proteins in the lens [29]. The extract increased the levels of glutathione and lens proteins accounting for the observed improved lens transparency in the treated rats.

The role of phytoconstituents in the prevention/treatment of age-related ocular disorders such as cataract has been widely reported [47]. In the preclinical stage, flavonoids have been shown to be protective against eye lens opacification by inhibiting glycoxidation [48, 49]. Other studies have proven that alkaloids can inhibit oxidative damage caused by reactive oxygen species such as H_2_O_2_ [50, 51]. The ability of the extract to attenuate cataract formation could therefore be attributed to the presence of these phytoconstituents in it.

The extract prevented or delayed selenite-induced cataract in rodents. This result is desirable clinically because a delay in cataract formation could prevent the visual impairment associated with this disease that could affect the independence of its victims [52]. It is stipulated that a delay in cataract formation can improve the lives of its victims by about 10 years, reducing their dependency on others [53]. Such a delay seen in the middle-dose treatment has the tendency to improve the lives of individuals especially the elderly for an appreciable timeframe.

The obvious impact of oxidative stress on selenite cataract is seen in how rapidly nuclear cataracts appear in a maximum of 5 days after sodium selenite administration. Phytochemicals such as tannins and flavonoids have antioxidant properties and mop up these reactive species [54]; hence, their presence in the extract may have been responsible for the preventive effect in the development of the senile cataract.

The cascade of biochemical processes that take place in the selenite cataract includes the reduction of proteins in general and the insolubilization of soluble proteins in the lens [55]. Proteins function as transport channels, and hence in selenite-induced cataract, there is the destruction of transport system in the lens [56, 57]. Alpha-A crystallin plays a chaperone role in the lens [58–60], while aquaporin 0 (AQP0), also known as major intrinsic polypeptide (MIP), is water channel in the lens [61]. The extract maintained significant levels of these markers in the lens accounting for the observed anticataract effect.

### 4.1. Conclusions

In conclusion, the aqueous extract of *A*. *africana* flowers can protect the lens from galactose-induced cataractogenesis while slowing down development of selenite-induced cataractogenesis in Sprague Dawley rats. The possible underlying mechanism may be partly explained by the decreased oxidative stress and the maintenance of high levels of aquaporin 0 and alpha-A crystallin in the lens. However, further studies need to be done to clarify its exact mechanism of anticataract activity.

## Figures and Tables

**Table 1:** Scoring stages of the crystalline lens in galactose-induced cataract in Sprague Dawley rats.

Grade	Level of opacity description	Score
	Clear lens with no vacuole	0
I	Clear lens with <3 vacuoles	1
II	Clear lens with >3 vacuoles	2
III	Vacuoles enveloped the whole lens surface	3
IV	Incomplete lens opacity	4
V	Total lens opacity	5

**Table 2:** Grading and scoring of lenses of Sprague Dawley rat pups with selenite-induced cataract.

Grade	Description	Score
	Clear lens	0
I	Distended lens fibres and subcapsular opacities	1
II	A nuclear cataract was observed in the lens, but distended fibres were still observable in the lens cortex	2
III	Intense nuclear cataract with perinuclear area opacity in the lens	3
IV	Complete opacity of the lens	4

**Table 3:** Results of the phytochemical screening of AAE.

	Tannins	Alkaloids	Flavonoids	Triterpenoids	Sterols	Saponins	Glycosides
AAE	+	+	+	+	−	+	+

**Table 4:** *In vitro* lens aldose reductase inhibitory effect of AAE with quercetin as positive control.

Treatment	Concentration (*µ*g/ml)	% inhibition	IC_50_ (*µ*g/ml)
AAE	50	9.96	12.12
	100	12.21	
	200	16.29	
	400	20.27	
	800	22	

Quercetin	50	32.17	102.6
	100	41.14	
	200	44.57	
	400	45.8	
	800	47	

**Table 5:** Various standard equivalents for the *A*. *africana* extract.

Test	AAE (*µ*g/g)
Total antioxidant capacity (ascorbic acid equivalent)	843.30
Total polyphenol content (gallic acid equivalent)	764.73
Total flavonoid content (quercetin equivalent)	408.90

## Data Availability

The datasets generated and/or analyzed during the current study are available from the corresponding author on reasonable request.

## Additional Points

*Highlights*. (i) The aqueous flower extract of *Aspilia africana* alleviates experimentally induced age-related and diabetic cataract in rodent models. (ii) The extract was able to preserve markers of lens transparency, such as aquaporin 0 and alpha-A crystallin, in age-related cataracts in rats. (iii) The extract increased total lens proteins and lens glutathione levels in the rodent diabetic cataract model.
